# Pancreatic metastasis from renal cell carcinoma presenting as gastrointestinal hemorrhage: a case report

**DOI:** 10.1093/jscr/rjab368

**Published:** 2021-08-31

**Authors:** Satoshi Matsui, Hiroaki Ono, Daisuke Asano, Yoshiya Ishikawa, Hiroki Ueda, Keiichi Akahoshi, Kosuke Ogawa, Atsushi Kudo, Shinji Tanaka, Minoru Tanabe

**Affiliations:** Department of Hepatobiliary and Pancreatic Surgery, Graduate School of Medicine, Tokyo Medical and Dental University, Tokyo, Japan; Department of Hepatobiliary and Pancreatic Surgery, Graduate School of Medicine, Tokyo Medical and Dental University, Tokyo, Japan; Department of Hepatobiliary and Pancreatic Surgery, Graduate School of Medicine, Tokyo Medical and Dental University, Tokyo, Japan; Department of Hepatobiliary and Pancreatic Surgery, Graduate School of Medicine, Tokyo Medical and Dental University, Tokyo, Japan; Department of Hepatobiliary and Pancreatic Surgery, Graduate School of Medicine, Tokyo Medical and Dental University, Tokyo, Japan; Department of Hepatobiliary and Pancreatic Surgery, Graduate School of Medicine, Tokyo Medical and Dental University, Tokyo, Japan; Department of Hepatobiliary and Pancreatic Surgery, Graduate School of Medicine, Tokyo Medical and Dental University, Tokyo, Japan; Department of Hepatobiliary and Pancreatic Surgery, Graduate School of Medicine, Tokyo Medical and Dental University, Tokyo, Japan; Department of Molecular Oncology, Graduate School of Medicine, Tokyo Medical and Dental University, Tokyo, Japan; Department of Hepatobiliary and Pancreatic Surgery, Graduate School of Medicine, Tokyo Medical and Dental University, Tokyo, Japan

**Keywords:** renal cell carcinoma, pancreatic metastasis, bleeding, transarterial embolization

## Abstract

In some patients with metastatic renal cell carcinoma to the pancreas, gastrointestinal hemorrhages occur, but because of the rarity of this condition, treatment strategies have not been established. A 71-year-old man who had undergone a nephrectomy for renal cell carcinoma (RCC) went to a hospital in a state of shock. Computed tomography revealed a hypervascularized tumor in the head of the pancreas, suggesting metastatic RCC. Upper endoscopy revealed bleeding in the duodenum due to tumor invasion. An emergency angiogram showed that the tumor received its blood supply mainly from the gastroduodenal artery. Transarterial embolization (TAE) of the gastroduodenal artery was performed and bleeding was controlled. Two months after TAE, elective pancreaticoduodenectomy was performed. The patient currently continues to undergo outpatient follow-up 2 years later without recurrence. TAE was very effective in controlling the acute phase of severe gastrointestinal hemorrhage from pancreatic metastasis of RCC.

## INTRODUCTION

In some patients with metastatic renal cell carcinoma (RCC) to the pancreas, gastrointestinal hemorrhages occur [1], but because of the rarity of this condition and the limited number of cases in the literature, treatment strategies for gastrointestinal hemorrhage caused by metastatic RCC to the pancreas have not been established [2]. We present a case of severe gastrointestinal hemorrhage causing the patient to enter a state of shock due to metastatic RCC to the pancreas.

## CASE REPORT

A 71-year-old man had undergone a left nephrectomy for RCC 17 years earlier. The patient had not shown any signs of recurrence but was admitted to hospital for melena with signs of shock. Contrast computed tomography (CT) revealed a hypervascularized tumor in the head of the pancreas. This suggested that metastatic RCC was the most likely diagnosis. On esophagogastroduodenoscopy, bleeding at the descending part of the duodenum was observed, which was thought to be due to duodenal invasion of the tumor in the pancreatic head. It was not possible to control the bleeding endoscopically and the patient was therefore transferred to our hospital for specialist treatment.

**Figure 1:**
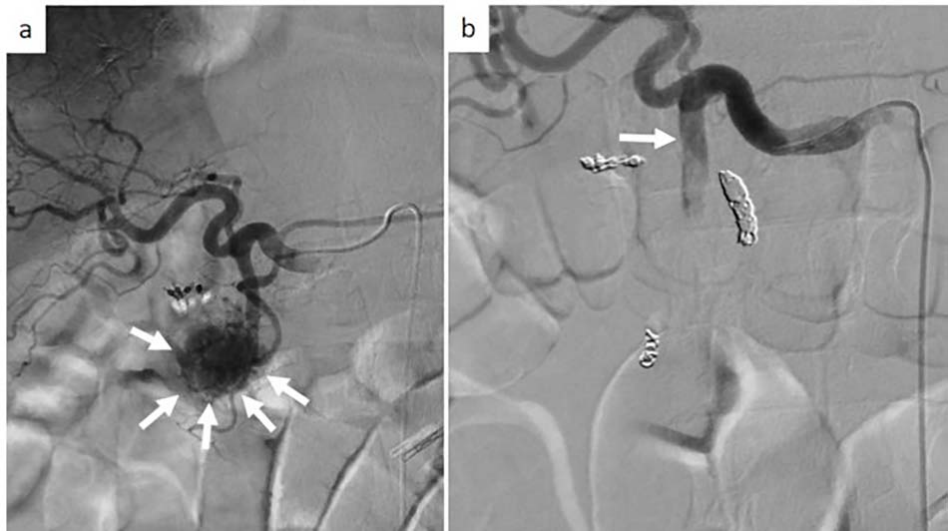
Emergency angiography; (**a**) emergency angiography showing the hypervascular tumor in the pancreas head (white arrow); (**b**) embolization of the gastroduodenal artery (white arrow).

On examination, the patient had conjunctival pallor, blood pressure: 147/79 mmHg after transfusion and pulse: 95 beats per minute. Laboratory blood tests revealed a hemoglobin level at 7.8 g/dl. We decided to perform an emergency angiography which revealed a highly vascularized tumor at the pancreas head (Fig. 1a). The tumor was supplied with blood from a branch of the gastroduodenal artery. No extravasation of contrast medium was observed. Embolization of the anterior superior pancreatoduodenal artery and the right gastric epiploic artery was performed, and then also of the gastroduodenal artery, the right gastric epiploic artery, and the gastroduodenal artery. Angiography thereafter confirmed that all feeder arteries of the tumor had been embolized (Fig. 1b). Gastrointestinal hemorrhaging ceased after embolization. The patient did not complain of any abdominal pain after embolization and his medical condition was stable.

**Figure 2:**
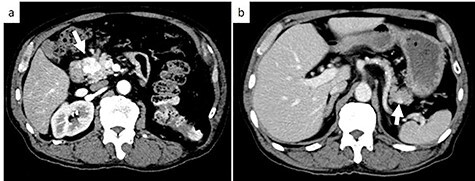
Axial contrast-enhanced CT images; (**a**) CT showing a 25-mm well-defined hypervascular heterogeneous lesion in the pancreas head; (**b**) CT showing a 9-mm well-defined hypervascular heterogeneous lesion in the pancreas tail. White arrows indicate the tumor in the pancreas head and tail.

Subsequent contrast CT revealed two contrast-enhanced nodules in the pancreas head and tail (Fig. 2) showing that the tumor which caused the bleeding had a reduced uptake of contrast agent. Esophagogastroduodenoscopy showed a submucosal tumor in the descending part of the duodenum. Positron emission tomography-CT revealed a lesion with weak fluorodeoxyglucose uptake at the pancreas head (maximum standardized uptake value: 2.1). There were no lesions with fluorodeoxyglucose uptake in the tail of the pancreas or other organs.

On the basis of these tests, we suspected that both of the tumors were due to multiple metastases of RCC to the pancreas. We performed pancreatoduodenectomy (PD) for the pancreas head tumor and partial pancreatectomy for the pancreas tail tumor. The patient was discharged on post-operative Day 20 without any complications. During 30 months of follow-up, there was no evidence of local recurrence or distant metastasis.

**Figure 3:**
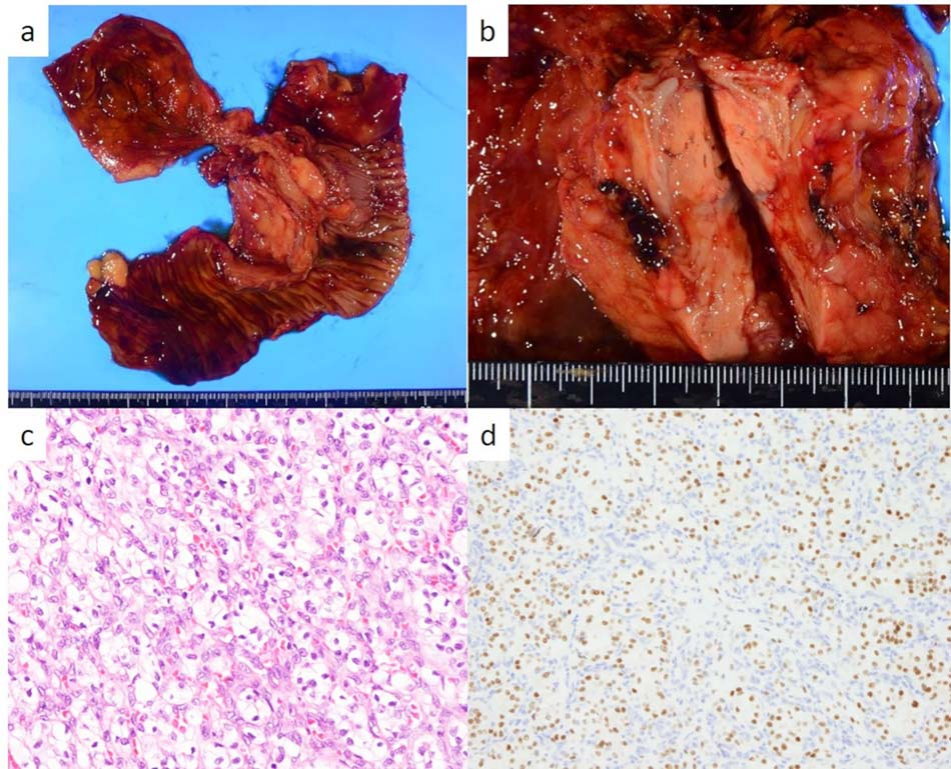
Resected specimen and pathological findings of the pancreatic head tumor; (**a**, **b**) a yellow 38-mm solid tumor in the pancreatic head protruding into the duodenum; (**c**) abundant nests of large clear cells (hematoxylin and eosin ×400); (**d**) immunohistological positivity for PAX8 (PAX stain ×200).

**Figure 4:**
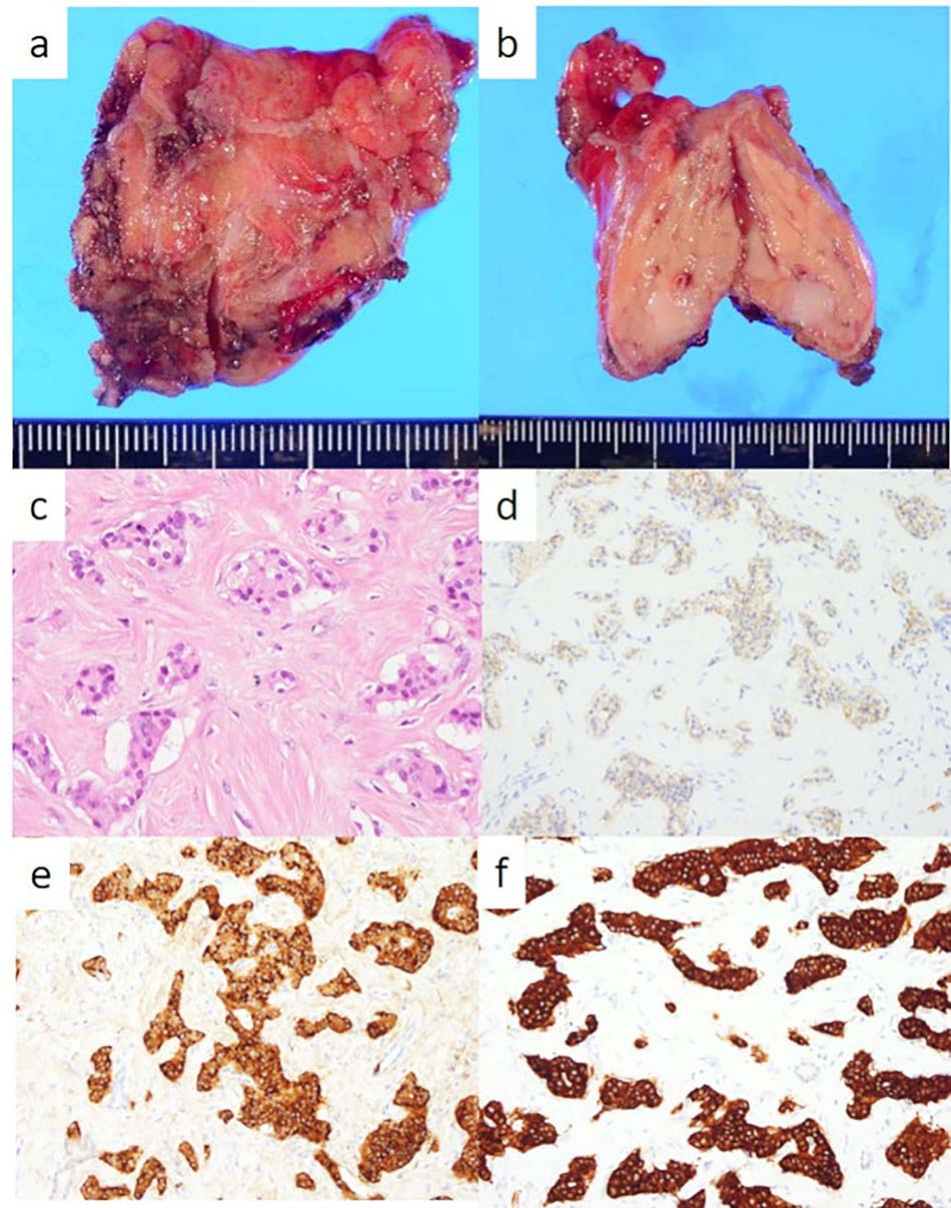
Resected specimen and pathological findings of the pancreatic tail tumor; (**a**, **b**) a white 6-mm solid tumor in the pancreatic tail; (**c**) abundant nests of large clear cells (hematoxylin and eosin ×400); (**d**) immunohistological positivity for CD56 (CD56 stain ×200); (**e**) immunohistological positivity for chromogranin A (chromogranin A stain ×200); (**f**) immunohistological positivity for synaptophysin (synaptophysin stain ×200).

The head tumor was a yellow 38 × 20 × 14 mm solid tumor with capsule, protruding into the duodenum (Fig. 3). Histopathology revealed abundant nests of large clear cells with nested architecture and prominent vasculature (Fig. 3). Immunohistochemistry staining showed positive immunoreactivity for PAX8, suggesting metastasis of RCC (Fig. 3). By contrast, the tail tumor was a white 6 × 6 mm solid tumor with defined cell borders. Histopathology revealed atypical cells with hyperchromatic nuclei in the nests (Fig. 4). Immunohistochemistry showed the following pattern: CD56 (+), chromogranin A (+), synaptophysin (+), Ki67 0.8% (4/489) (Fig. 4). Thus, the tail tumor was diagnosed as a pancreatic neuroendocrine tumor, G1.

## DISCUSSION

Among cancers occurring in the pancreas, metastatic lesions are rare, comprising ~2–3% of all pancreatic malignancies [3, 4]. The most common distant metastases to the pancreas are derived from RCC [1, 4]. Sellner *et al*. reported that 35% of the metastases did not cause any symptoms [5]. In patients with metastatic RCC to the pancreas, symptoms such as pain (20%), gastrointestinal bleeding (20%), weight loss (9%) and jaundice (9%) were common [5].

The effectiveness of transarterial embolization (TAE) for unresectable primary or metastatic disease in RCC patients has been established [6]. However, only a few studies have reported the effectiveness of TAE for bleeding due to metastatic RCC to the pancreas [2]. This may be because TAE is avoided due to risks of ischemic complications [2]. Although the upper gastrointestinal tract usually has a rich collateral blood supply, previous studies have shown that ischemic complications occur in 7–16% of cases [7, 8]. A recent systematic review on embolization for non-variceal upper gastrointestinal tract hemorrhage found a mean clinical success rate of 67%, while the mean rebleeding rate, mean complication rate and mean 30-day mortality were 27, 6 and 8%, respectively [9]. Based on these studies, a recent meta-analysis showed that TAE is a safe and effective procedure and could be a viable option for the first-line therapy of refractory non-variceal upper gastrointestinal tract hemorrhage [10].

Emergency PD, which is performed for bleeding, is accompanied by a higher frequency of complications than elective PD [11, 12]. Tsai *et al*. reported overall complication and surgical mortality rates for emergency PD of 83.9 and 19.4%, respectively. Propensity score matching further indicated a significantly higher surgical mortality of emergency than elective PDs (19.4 vs. 3.2%) [12]. These investigators proposed that a possible explanation for the higher morbidity and mortality of emergency PD is that emergency surgery is always associated with a lesser degree of preparedness than elective surgery [12]. The treatment strategy of TAE followed by subsequent elective pancreatectomy may be effective both in acute and chronic phases of hemorrhagic events due to RCC.

When metastasis is limited to the pancreas, complete resection can result in a 5-year survival rate of 43–88% [13]. Analyzing 11 studies with five or more patients, which addressed pancreatic metastasis from RCC, Reddy *et al*. noted that in 7 studies (64%), the 5-year survival rate from the time of pancreatic resection was 80% [14]. A better prognosis after pancreatic metastasectomy can be achieved.

Pancreatic metastasis from RCC may present many years after resection of the primary RCC and it can present as upper gastrointestinal bleeding. TAE and elective pancreas resection may be effective both in acute and chronic phases of hemorrhagic events due to RCC.
